# Assessment of Replicability and Efforts to Refine an Operant Conditioning Procedure for Larval Zebrafish

**DOI:** 10.3390/ani14243684

**Published:** 2024-12-20

**Authors:** Christian Agrillo, Eleonora Rovegno, Marco Dadda, Cristiano Bertolucci, Angelo Bisazza

**Affiliations:** 1 Department of General Psychology, University of Padova, 35122 Padua, Italy; marco.dadda@unipd.it (M.D.); angelo.bisazza@unipd.it (A.B.); 2 Padua Neuroscience Center, 35131 Padova, Italy; 3 Department of Life Sciences and Biotechnology, University of Ferrara, 44121 Ferrara, Italy; rvglnr@unife.it (E.R.); cristiano.bertolucci@unife.it (C.B.)

**Keywords:** discrimination learning, animal cognition, behavioral neuroscience, replication study

## Abstract

Zebrafish have emerged as prominent model organisms in neurobiological research. Although most studies are performed on embryonic and larval stages within the first week post-hatching, tools for assessing cognitive functions during these early developmental stages remain limited. An appetitive conditioning paradigm has been recently developed that demonstrates discrimination learning within days of hatching and holds promise for the assessment of perception, memory, and executive functions. Despite its potential, the utility of this paradigm is hindered by certain limitations and confounding factors that may undermine its replicability. To address these concerns, we conducted a replication of the experiment in a separate laboratory, which revealed partial replicability and confirmed the drawbacks identified in the original study. Subsequently, we explored two modifications to the procedure in an effort to overcome these limitations. While both experiments demonstrated significant stimulus–response associations, the outcomes were unsatisfactory as performance in both cases fell well below that of the original procedure.

## Simple Summary

Larval zebrafish are increasingly used as a model for studying the neurobiology of cognition, but reliable methods for assessing cognitive functions such as learning and memory in their early developmental stages remain scarce. A recently developed operant conditioning procedure allows newly hatched larvae to associate a specific stimulus with a food reward, providing a tool to investigate diverse aspects of cognition. However, this method also presents some limitations that could hinder its adoption as a standard procedure across research laboratories. In our study, we replicated the procedure in a separate laboratory, achieving only partial success. Two further experiments were conducted to optimize the methodology. While both modified procedures con-firmed a stimulus–response association in newborn larvae, neither achieved the performance levels reported by the original research. Our findings underscore the importance of validating and refining behavioral paradigms to ensure their robustness and ease of reproduction across laboratories. This work contributes to establishing reliable methods for exploring cognitive development in zebrafish, with potential implications for broader neurobiological research.

## 1. Introduction

The zebrafish model is rapidly advancing numerous areas of basic and translational research, including toxicology, drug discovery, molecular genetics, genomic research, developmental biology, and the study of human diseases (reviewed in [1,2,3]). In recent years, it has also emerged as a key model in neurobiological research, quickly matching—and in some cases surpassing—the importance of traditional models such as rodents and *Drosophila* [4,5]). Most research on zebrafish neurobiology is performed on embryos and larvae in the first days after hatching. Larvae can be produced in large numbers, allowing large-scale generation of mutant or transgenic lines and chemical drug screening [6,7]. In addition, due to their transparency, the whole brain activity can be recorded at cellular resolution [8]. Significant advancements have been made in the development of larval zebrafish models to explore a spectrum of neuropathologies, encompassing neurodevelopmental and neurodegenerative disorders (reviewed in [9,10,11]) such as Alzheimer’s disease [12], along with investigations into the re-myelination process in conditions such as multiple sclerosis and brain injuries (reviewed in [13]).

In many of these cases, in addition to examining the functioning of the nervous system at the neural level, it would be useful to directly assess cognitive functions. In murine models and Drosophila, tools to study cognitive functions such as visual perception, learning and memory, and executive functions have been available for years [14,15] and similar tools have been developed for juvenile and adult zebrafish [16,17,18]. Attempts have also been made to adapt assays developed for traditional model species or adult zebrafish to newly hatched larvae. However, in most cases, these efforts have proven to be problematic or subject to significant limitations, severely hindering research in many fields.

Best and collaborators [19] developed a learning assay based on habituation for 7 days post-fertilization (dpf) larval zebrafish, which consists of measuring changes in the startle response to a series of acoustic stimuli. However, this method only allows for study of a particular class of learning mechanisms—non-associative learning—and evaluates responses to a limited range of very simple stimuli. Aizenberg and Schuman [20] developed a classical conditioning paradigm in which 6 to 8 dpf larvae, immobilized in agarose, are conditioned by repeatedly pairing a visual stimulus (light flashes) with a touch to their tail. The immobilization of the larvae in agarose greatly limits the tasks that can be administered with this procedure, and furthermore, this method does not allow for the use of complex stimuli or the study of simultaneous discrimination, which are fundamental to many cognitive tests. Another aversive learning procedure can partially circumvent some limitations of the previous paradigm [21]. From 7 dpf, larvae can be trained to avoid the darker side of their tank through the administration of a mild electric shock but it is not known whether they can be conditioned using more complex stimuli. Other methods have been developed in recent years, yet their applications remain limited to highly specific areas of study [2,22].

Bruzzone and collaborators [23] attempted to develop a general procedure that could be employed to study various aspects of visual perception and cognition. They adapted a classic memory test originally designed for rodent research, the one-trial memory test, for use with larval zebrafish. In this test, a rat is exposed to a stimulus for a few minutes and, after a delay, presented again with the familiar stimulus alongside a novel one. If the rats discriminate between the two stimuli, they spend more time exploring the novel one, which indicates memory retention [24]. Larval zebrafish were found capable of discriminating between two objects or two printed geometric figures. However, unlike rats, zebrafish, as other teleosts, can respond to a novel stimulus by either approaching or moving away from it. The type of response depends on various factors such as neophobia or anxiety-like levels, which, in turn, are influenced by numerous variables including age, sex, type of stimuli used, or previous experience. Since memory measurement in this test relies on a simple response (percentage of time spent with either of the two stimuli), it is evident that this procedure is strongly confounded by various factors when applied to fish. For example, a recent study used this procedure in an attempt to determine whether rearing larval zebrafish in an enriched environment affects their cognitive functions, particularly memory [25]. While it was found that only larvae kept in an enriched environment spent significantly more time near the novel stimulus, the research showed that this was not determined by cognitive differences, but most likely by the fact that rearing larvae in an enriched environment reduces their neophobic response.

The development of these procedures has partially advanced research on fundamental forms of learning and memory in larval zebrafish. However, investigating higher-order cognitive processes in animal models, such as spatial learning, cognitive flexibility, problem solving, working memory, or inhibitory control, has historically relied on appetitive conditioning paradigms (e.g., [26]). Until very recently, it remained unclear whether newly hatched larvae possess the capacity for appetitive learning or whether such paradigms could be adapted for early developmental stages.

### The Target Study

Santacà and colleagues [27] have recently developed a discrimination learning procedure based on an appetitive operant paradigm that can be administered to larval zebrafish in the first week after hatching. The paradigm is very similar to that employed in research with rodents, primates, and adult zebrafish, and has the potential to be used for measuring a variety of cognitive functions, including visual perception, learning and memory, and executive functions [27,28,29]. Larvae are trained for five days (from 8 dpf to 12 dpf) with two reinforcement tests per day in an apparatus consisting of two chambers connected by a small hole (10 mm in diameter). At the beginning of each trial, the two stimuli to be discriminated are introduced, one in each chamber, and after a delay, food was administered in the chamber containing the stimulus to be reinforced. The degree of learning was assessed prior to reward administration by measuring the percentage of time the larvae spent in the chamber containing the reinforced stimulus during the interval between stimulus introduction and food delivery. In this paradigm, the function of the narrow passage is to prevent food particles from passing into the chamber containing the unreinforced stimulus and to minimize the diffusion of food odor, which could otherwise act as secondary reinforcement with the incorrect stimulus.

**Figure 1:**
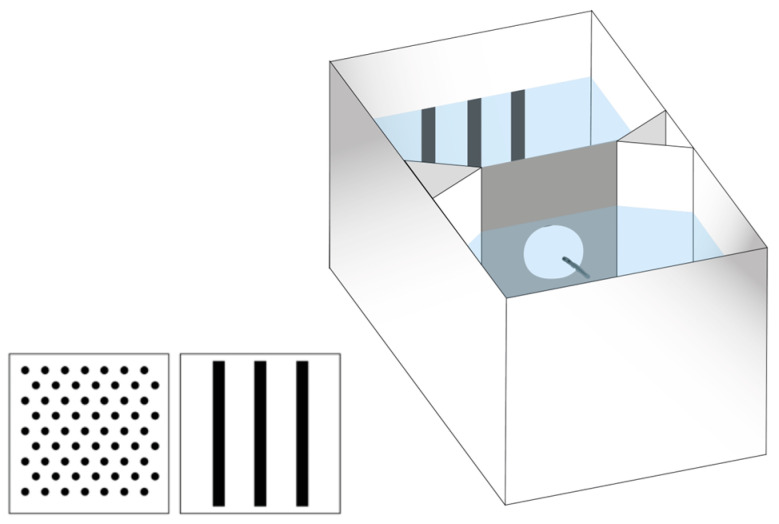
Experimental apparatus (**right**) and stimuli (**left**) used in Experiment 1.

In the above-mentioned study, during the first two days, subjects were trained collectively, as a pilot experiment showed that larvae trained individually from the beginning hardly learn to navigate the hole separating the two chambers. To test the new operant conditioning procedure, in a first experiment, larvae were required to discriminate two visual patterns that differed for a number of features (e.g., size, shape, and number of items: see Figure 1) to maximize their discriminability. After 5 days and 10 reinforced trials, subjects spent twice as long in the compartment with the positive stimulus, indicating a robust stimulus–reward association. An analysis of individual days showed that by the second day of training, preference for the reinforced stimulus was already established. In four subsequent experiments, aimed to test the versatility of the method, they found that larvae could learn to discriminate stimuli that differ only for color or for shape. They could also learn to discriminate a figure from its mirror image and from its 90° rotated version, although with much lower accuracy.

There are, however, several limitations and potential confounding factors inherent in this conditioning procedure. Firstly, some subjects exhibited frequent movements between chambers, while others showed minimal movement, registering zero passages in various trials. For the latter group, due to their limited mobility, if they happened to start a trial on the side of the incorrect stimulus, they could plausibly miss the opportunity to receive reinforcement. Therefore, it was decided to exclude subjects whose total number of passages between compartments in the six individual trials was less than 10 (1.7 on average per trial). In this way, however, more than 40% of the subjects were eliminated from the analyses. In addition, even the more mobile subjects probably experienced trials with no passages in some cases, resulting in variations in the number of reinforced trials received among subjects admitted to the final sample.

This practice introduces a selection bias among subjects that is likely non-random. Subjects with many passages may differ from those with few, potentially varying in locomotor activity, exploratory tendency, or boldness, which could introduce significant confounders when comparing different treatments. For instance, pre-experimental experiences or the administration of certain drugs have been shown to influence activity and anxiety-like behavior in young larvae [30,31]. In a recent study, larvae reared in an enriched environment exhibited higher activity levels, faster swimming, and more frequent movement between compartments compared to controls raised in a bare environment [32].

A second concern involves an unusually high mortality rate observed in this study, nearly 30%, compared to the usual rate of less than 5% found in other experiments with larvae of similar age in our laboratories [25,32,33]. The reason for this mortality may be related to the previously mentioned issue. The two meals provided per day are typically sufficient for larvae of this age. However, some larvae, due to their poor inter-compartment passages, may not have reached the correct compartment in time for the food to be edible, potentially leading to starvation. High experimental mortality could also introduce confounding factors, as surviving larvae may possess different genetic, physiological, or behavioral characteristics than those that died.

A procedure that excludes a large portion of subjects can still serve to explore the boundaries of cognitive abilities in young larvae, but is impractical as a cognitive test in translational research. For instance, mortality and limited mobility pose challenges when applying this procedure to mutant lines, which are frequently associated with reduced fertility and survival rates. Moreover, the substantial selection bias observed in subjects completing the experiment indicates that introducing confounding variables is highly probable, diminishing the replicability of research conducted using this method.

On the other hand, many procedures commonly used to study animal cognition require the exclusion of a substantial proportion of subjects from the experiment, for instance because they are inactive or uncooperative (e.g., [34,35]). In other cases, the presence of significant confounding factors is well documented [36,37,38]. Nevertheless, these methods continue to be employed either because they are robust to such methodological limitations or because no valid alternatives exist for measuring the targeted cognitive function.

The first experiment of this study aimed to assess the robustness of this procedure by measuring its replicability. We replicated Experiment 1 from the previous study [27] in another laboratory, following the exact procedure and utilizing the same equipment and stimuli. Since we observed only partial reproducibility of the procedure and encountered similar issues with larval survival and mobility, in two additional experiments, we explored modified procedures to address these limitations. In one approach, we widened the hole connecting the two halves and enlarged the apparatus to increase the distance between the points where food was released. In the other, we further expanded the passage between the two halves while implementing a solution to prevent food or its odor from reinforcing the negative stimulus.

## 2. Materials and Methods

### 2.1. Experiment 1

Experiment 1 was conducted in the Behavioural Biology laboratory of the University of Ferrara and was a replication of Experiment 1 of the target study [27] with only minor variations due to the adaptation to the different laboratory. This is a paradigm of appetitive learning. Larvae were trained over a five-day period, with two trials per day, in a two-chamber apparatus. During each trial, the two stimuli to be discriminated were introduced, one in each chamber, and after a delay, a food reward was delivered near the positive stimulus. In our procedure, stimulus–reward association was evaluated by measuring the percentage of time larvae spent in the chamber containing the reinforced stimulus during the period preceding food delivery.

#### 2.1.1. Subjects

In this experiment, we tested 48 zebrafish larvae. A pilot experiment conducted in the original study [27] estimated a minimum sample size of 10 to achieve statistical significance (*p* = 0.05) with 90% power in a two-tailed *t*-test. Considering the mortality rate reported in that study, we anticipated that the final sample size would comfortably exceed this threshold. Throughout this manuscript, we followed the standard age classification used in zebrafish research, starting from the day of fertilization and expressed as days post-fertilization (dpf) e.g., [39].

The experimental subjects belonged to an outbred, wild-type population kept in the fish facility at University of Ferrara. The eggs were collected in several Petri dishes (10 cm Ø, 1.5 cm high) in a solution of E3 1× (5 mM NaCl, 0.17 mM KCl, 0.33 mM CaCl_2_, 0.33 mM MgSO_4_) and methylene blue (0.0016 g/L). Hatched larvae were then transferred in Petri dishes in fish water solution 1 × (0.5 mM NaH_2_PO_4_·H_2_O, 0.5 mM Na_2_HPO_4_·H_2_O, 1.5 g Instant Ocean, 1 L deionized H_2_O). Before the experiment, they were housed in the same room at a density of approximately 30 larvae per Petri dish, which were maintained at a temperature of 28.5 ± 1 °C and illuminated according to a 14:10 h light:dark cycle. Zebrafish larvae were fed from the age of 6 dpf twice a day with dry food (SERA Micron Nature, particle size range 50–150 µ) containing zooplankton (18% krill) and phytoplankton (51% spirulina). The same food was used as a reward during training.

#### 2.1.2. Apparatus

We used the same equipment employed in the previous study [20] which was transferred from the University of Padova. Two different apparatuses were used. For the familiarization phase and the initial group training, we used an hourglass-shaped tank (12 × 4.8 cm and 4 cm high) consisting of two identical compartments separated by a grey plastic panel (3 × 3.2 cm) with a central hole (1 cm Ø) allowing larvae to move between compartments (Figure 1). Individual training was conducted in a similar but smaller tank (7 × 4 cm and 4 cm high) filled with 3.5 cm of fish water 1×. To prevent the subjects from seeing their surroundings, the training apparatuses were placed in a white plastic box (containing four tanks for collective training or twelve for the individual training) and lit by two led strips placed symmetrically along the major axis of the plastic boxes. The room was kept in semi-darkness.

We used the same stimuli used for Experiment 1 of the previous study [27], two patterns that differed for multiple cues (orientation and spatial frequency of the pattern, size, shape, and number of the single items), but had an equal proportion of black and white surfaces (Figure 1).

#### 2.1.3. Procedure

The experiment consisted of three phases: a preliminary two-day familiarization phase (6 to 7 dpf), a two-day group training phase (8 to 9 dpf), and a three-day individual training phase (10 to 12 dpf).

Familiarization and group training phase: Six dpf larvae were moved using a Pasteur pipette in the familiarization tanks, in which no grey panel was inserted. The morning of the subsequent day, the panel with the hole was inserted to habituate larvae to pass through the hole and move between the two compartments. During these two days, subjects were fed twice a day, with the food delivered in both compartments. Group training began when larvae were 8 dpf and lasted two consecutive days. Half subjects were trained on one stimulus and half on the other. Each day, we administered two reinforced trials, in the morning and in the afternoon (reward administered at 10:30 and 15:00 respectively).

Individual training: One hour after the afternoon trial of the second day of group training, larvae were moved to the individual test tank and let undisturbed until the next morning. The test phase lasted three more days, during which we administered two reinforced trials per day with the same temporal schedule of the group training phase. To avoid any cueing effect due to food residuals from the previous trial, each subject was gently pipetted and moved to an identical clean tank at the end of each trial.

Trial: The procedure for a trial was the same for group and individual training. The two stimuli were introduced in the apparatus 90 min prior to reward administration and placed against the tank’s two short walls. During this time, we video-recorded the movement of subjects in the apparatus (only for the individual training phase). These recordings were subsequently analyzed to measure the preference for the reinforced stimulus over the other. Food was then delivered near to the reinforced stimulus. The two stimuli remained during feeding and were removed 90 min from the reward delivery. To avoid side biases, stimuli were swapped between compartments every other trial. The position was reversed between the morning and afternoon trials for half of the replicates and after the end of the second trial for the other half.

Overall, each subject performed ten reinforced trials, four during the group training and six during the individual training. Only these latter were scored and analyzed. As in the previous study, after the end of the experiment, the larvae were kept for a further 24 h in their tank and fed twice a day as before. Those that died during the experiment or in this post-experimental period were not considered in the analysis.

#### 2.1.4. Data Collection and Statistical Analysis

Videos were analyzed offline using a custom software (Ciclic Timer 1.3). For each of the six individual trials, we scored time spent by each larva in the compartment with the reinforced stimulus and in the compartment with the non-reinforced stimulus and the number of passages between the two compartments. Statistical analyses were performed using R (version 4.4.2; the R Foundation for Statistical Computing, Vienna, Austria, https://www.r-project.org). The measure of performance was the proportion of time spent in the compartment with the positive stimulus during the 90 min period preceding food delivery. We used one-sample *t*-tests to compare this measure with chance level (0.50). Significance was set at *p* < 0.05. We analyzed the subjects’ performance using generalized linear mixed-effects models. To assess whether performance improved across trials, we included trial number (1–6) as a fixed effect. Additionally, we included larvae mobility (number of passages between compartments) and the reinforced stimulus pattern (stripes or dots) as fixed effects, while individual ID was included as a random effect.

### 2.2. Experiment 2

In this experiment, we adopted essentially the same procedure as in Experiment 1, with a few modifications. Specifically, the passage between the two compartments was widened more than ten-fold to increase the likelihood of larvae moving between them. Additionally, the testing apparatus was elongated to further separate the two stimuli to be discriminated, minimizing the risk of food or its odor spreading into the vicinity of the non-reinforced stimulus.

#### 2.2.1. Subjects

The subjects of this experiment were 46 wild-type zebrafish larvae that originated from an outbred stock maintained in laboratory of the Department of General Psychology, University of Padova. Eggs and hatched larvae were housed in Petri dishes (10 cm Ø, h:1.5 cm) in a solution of fish water 1× (0.5 mM NaH_2_PO_4_·H_2_O, 0.5 mM Na_2_HPO_4_·H_2_O, 1.5 g Instant Ocean, 1 L deionized H_2_O) and methylene blue (0.0016 g/L). Following the indications of a recent study suggesting the provision of enrichment from hatching [32], we added 10 small LEGO^©^ bricks of varying shapes and colors to each Petri dish. Before the experiment, larvae were kept in the same room housing the experimental apparatus at a density of approximately 30 larvae per Petri dish, which were maintained at a temperature of 28.5 ± 1 °C and illuminated according to a 14:10 h light:dark cycle. Zebrafish larvae were fed from the age of 6 dpf twice a day with dry food (an admixture of GEMMA Micro 75 and shredded TetraMin flakes, particle size range: 50–150 µ). The same food was used as a reward during training.

#### 2.2.2. Apparatus

The apparatus used for this experiment was the same hourglass shaped tank used for the familiarization and group training phase of Experiment 1. For this experiment, the central passage was devoid of the grey plastic panel, so the opening between the two compartments was 26 mm wide and 28 mm high (7.28 cm^2^). Due to surface tension, floating food particles tended to spread within seconds between the two compartments. To prevent this phenomenon, a small panel was inserted at the water surface, reaching up to two mm in depth. The apparatus was filled with a solution of fish water with methylene blue added at half the concentration used for the Petri dishes. Stimuli were the same of previous experiment and were positioned on the short wall at the two opposite extremities of the hourglass. The tanks were aligned on a table in a dark room and surrounded by an opaque plastic barrier to prevent the subjects from seeing outside. They were illuminated by two LED strips positioned 50 cm above the table surface. Cameras were placed above the table to record the experiments.

#### 2.2.3. Procedure

The day before the first training day, 7 dpf larvae were transferred from the Petri dishes the experimental tanks, one larva per tank. For five days, from 8 to 12 dpf, larvae received two conditioning trials per day for a total of 10 individual trials.

We used two slightly different procedures. Half of the subjects were tested with the A sub-procedure: stimuli were always present in the experimental apparatus from the moment in which larvae were moved, at 7 dpf, to the end of the 5 training days. Stimuli were removed only during cleaning process or for switching their position and reinserted just after. The remaining subjects were tested with the B sub-procedure. This was identical to A sub-procedure for the first day (7 dpf), during which subjects were allowed to familiarize with the two stimuli. Training was, however, similar to the individual training of Experiment 1, with stimuli introduced at the beginning of each trial and removed at the end.

We ran an equal number of replicates with the A and B sub-procedures and an equal number of replicates reinforcing the dots or the vertical bars. However, due to mortality, at the end of the experiment the number of subjects included in the sample were: 11 A sub-procedure—dots reinforced, 9 A sub-procedure—bars reinforced, 11 B sub-procedure—dots reinforced, 8 B sub-procedure—bars reinforced.

In this experiment we adopted a contingency approach that differed from that of Experiment 1 and from the study by Santacà and colleagues [27]. In previous experiments, food was delivered near the reinforced stimulus at the same time for all subjects, irrespective of the position occupied by each subject in the apparatus. Here, reinforcement was delivered only when the subject was in the compartment with the correct stimulus. Basically, at the beginning of each trial, the camera was turned on and, for B sub-procedure, we introduced the two stimuli. After 30 min, food was delivered, but only to those subjects who were in the compartment with the stimulus to be reinforced. The remaining subjects were checked every 5 min, and when they moved to the correct compartment, food was given to them as well. If a subject never entered the correct compartment within a 30 min period, reward was still administered, in the sector containing the positive stimulus. The position of the two stimuli was reversed between the morning and afternoon trials for half of the replicates and for the other half after the end of the second trial, as previously.

#### 2.2.4. Data Collection and Statistical Analysis

**Figure 2:**
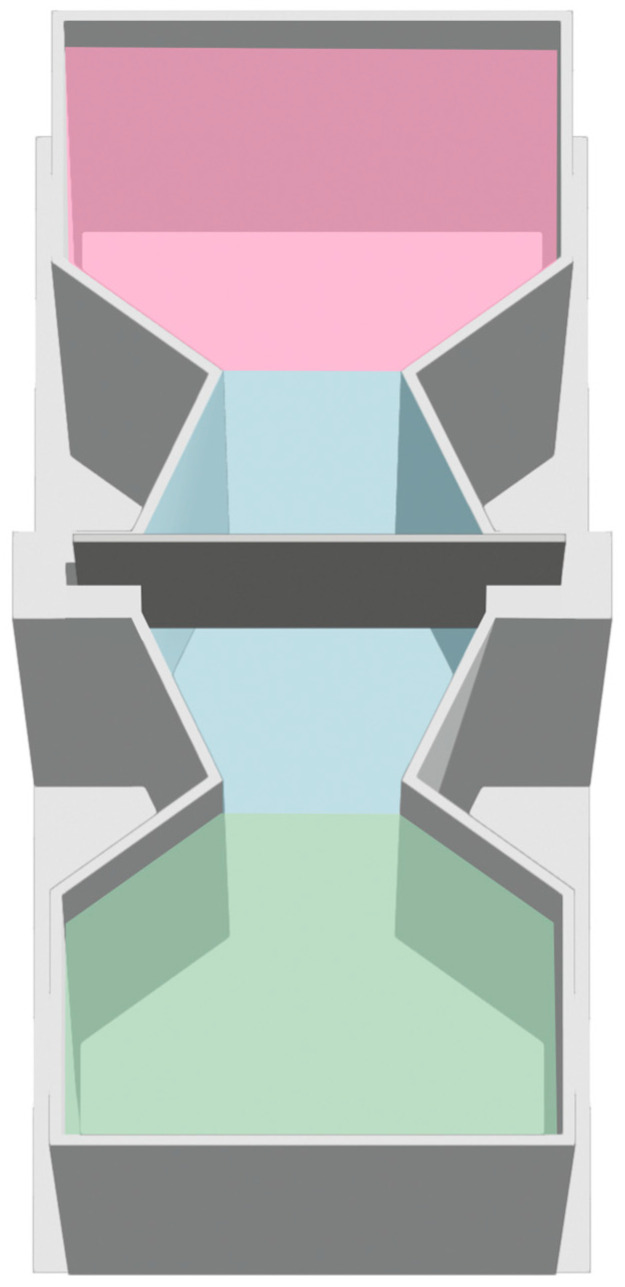
Virtual areas used to score stimulus preference in Experiment 2. Pink and green: stimulus areas, blue: no-choice area.

For data collection, the hourglass-shaped tank was divided in three virtual areas, two identical at the extremities, one containing the positive and one containing the negative stimulus, and a third with the connecting corridor in which there was no stimulus (no-choice area, Figure 2). Because of the shape of the apparatus and the division into three areas, it was not possible to obtain from the data a measure of passages between compartments similar to that of the previous trial. As a proxy for subject’s inter-compartment mobility, we used the percentage of trials in which there was at least one passage between one compartment and the adjacent. Due to a technical failure, data from 9 out of 390 trials were missing.

### 2.3. Experiment 3

In this experiment, a different way was sought to overcome the problem of reduced movement of the larvae between the two compartments. The apparatus was enlarged and provided with two large openings that presented no obstacles even at the water surface. As the flow of food over the water surface was no longer impeded in this apparatus, the solution implemented to avoid reinforcing the incorrect stimulus was to insert two identical stimuli of the reinforced type into both compartments at the time of reinforcement.

#### 2.3.1. Subjects

The subjects of the experiment were 32 larvae, maintained under the same conditions as in the previous experiment and kept until 3 dpf in Petri dishes (without enrichment with LEGO bricks). Zebrafish larvae were fed from the age of 6 dpf twice a day with dry food (an admixture of GEMMA Micro 75 and shredded TetraMin flakes, particle size range: 50–150 µ). The same food was used as a reward during training.

#### 2.3.2. Apparatus

**Figure 3:**
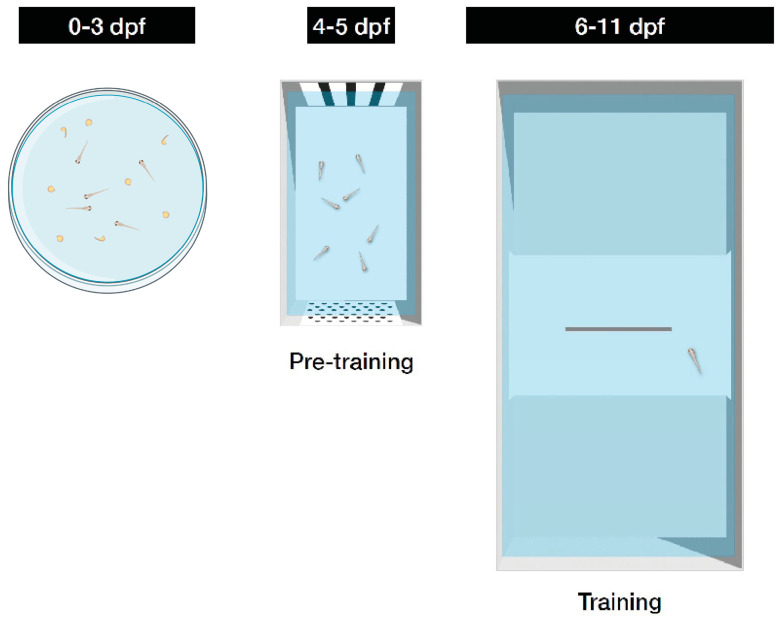
Apparatus used for post-hatching, pre-training and training periods in Experiment 3.

Two apparatuses were used in this experiment, both built with PLA using a 3D printer, one for pre-training phase and one for the individual training. In a pre-training familiarization phase, larvae were housed in rectangular white plastic tanks (4 × 7 cm) with the two stimuli used for the discrimination training phase placed along the short sides. The apparatus for individual training consisted of rectangular tanks (7 × 14 cm, 4 cm high, Figure 3). The two stimuli to be discriminated were placed on the short sides of the tank. A barrier was inserted at the midpoint of the longer side, preventing the subjects from seeing both stimuli simultaneously while allowing easy passage between compartments through two lateral openings. Each apparatus was then filled with a fish water solution with methylene blue to 50% of the concentration used for Petri dishes. The stimuli presented to the subjects were the same as those used in the two previous experiments. The tanks were aligned on a table in a dark room and surrounded by an opaque plastic barrier to prevent the subjects from seeing outside. They were lit by two LED strips positioned 50 cm above the table surface. Cameras were placed above the table to record the experiments.

#### 2.3.3. Procedure

As in previous experiments, the larvae were kept in Petri dishes until the morning of 4 dpf. Subsequently, the larvae were transferred with a pipette in groups of 15–20 to the pre-training apparatus with the two stimuli used for training placed on the short sides. Larvae are highly neophobic and respond with avoidance to unfamiliar stimuli. Accordingly, the subjects spent 2 days in this tank to familiarize with the stimuli to be discriminated, without receiving food. Note that, in the two previous experiments, familiarization with stimuli occurred at the beginning of the collective training phase. On the afternoon of 5 dpf, the larvae were individually transferred to each experimental apparatus, and training began on the morning of the following day (6 dpf) and concluded on 11 dpf. Compared to the two previous experiments, where fish started training at 7 dpf, training in this study began a day earlier. This change was necessary because at 5–6 dpf, the larvae start feeding, and if they were fed in the pre-training tank with the two stimuli, there would possibly develop an association with both stimuli. To ensure that the subjects finished at the same age as in the previous experiments, training was extended by one day, for a total duration of 6 days. Half of the experimental subjects were trained with the bar stimulus as positive and the other half with the dot stimulus.

**Figure 4:**
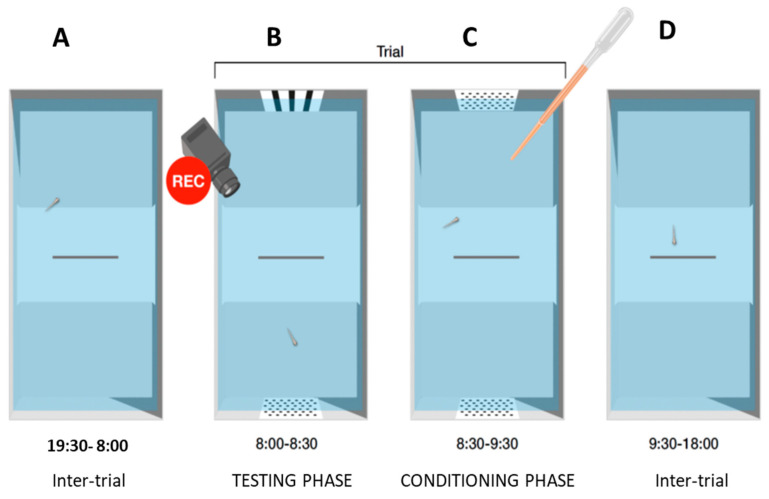
Timeline of a trial procedure (for the morning trial). (**A**) Subject spent inter-trial night period in a tank without stimuli. (**B**) At the start of the trial, two different stimuli were introduced, and preference was measured for 30 min. (**C**) Two different stimuli were removed and replaced with two identical stimuli (both of the reinforced type), then food was released in both compartments; larvae were left undisturbed for an hour. (**D**) Stimuli were removed for the duration of the next inter-trial period. A second trial was performed in the afternoon from 18:00 to 19:30 with an identical procedure.

Each trial consisted of a conditioning phase and a testing phase. On each of the 6 days of the experiment, the first test phase began at 8:00 a.m., with the introduction of the two stimuli to be discriminated, positioned centrally along the short side. After 60 s to allow the subjects to acclimate, recording began for 30 min. At the end of the test phase, at 8:30 a.m., the stimuli were removed and the conditioning phase began. This involved introducing a new pair of identical stimuli, both of the type reinforced for that subject. After a two-minute wait, a small amount of food for larvae was released in both sectors of the apparatus (Figure 4). The reinforcement phase lasted for 1 h, after which the stimuli were removed. After another hour, the excess degraded food, now aggregated into small masses, was removed from the surface with a pipette. This procedure was then repeated in the second trial of the day, which began at 6:00 PM. The position of the stimuli was reversed between the short sides between the morning and afternoon trials.

#### 2.3.4. Video Analysis

To determine the time spent in the sector with the reinforced stimulus and the sector with the non-reinforced stimulus, we analyzed the video recordings using BORIS (Behavioral Observation Research Interactive Software, version 8.27; University of Turin, Turin, Italy [40]). Using the keyboard to mark the moment when the subject changed compartments, the software calculated the total time spent in each sector of the apparatus, from which a preference index was calculated as the proportion of time spent in the sector with the reinforced stimulus.

## 3. Results

### 3.1. Experiment 1

A total of 24 out of 48 subjects died (50.0% mortality), 23 during the experiment and one after its end. This percentage is significantly higher than in the previous study (Santacà et al. [27]: data from all five experiments; 149 subjects, 106 surviving, 43 dead, mortality 28.8%; chi-square = 7.230, *p* = 0.007). During each 90 min period, larvae moved between compartments on average 1.36 ± 1.94 times (range 0–3.333).

Of the twenty-four surviving larvae, eight subjects (33.3%), moved 10 or more times between compartments in the total of six trials (the criterion adopted in previous study), and sixteen subjects less than ten times. The proportion of inactive subjects is almost twice the proportion observed in previous study (Santacà et al., [27]: 60/106, 56.6%), but the difference does not reach significance (chi-square = 3.366, *p* = 0.0665).

**Figure 5:**
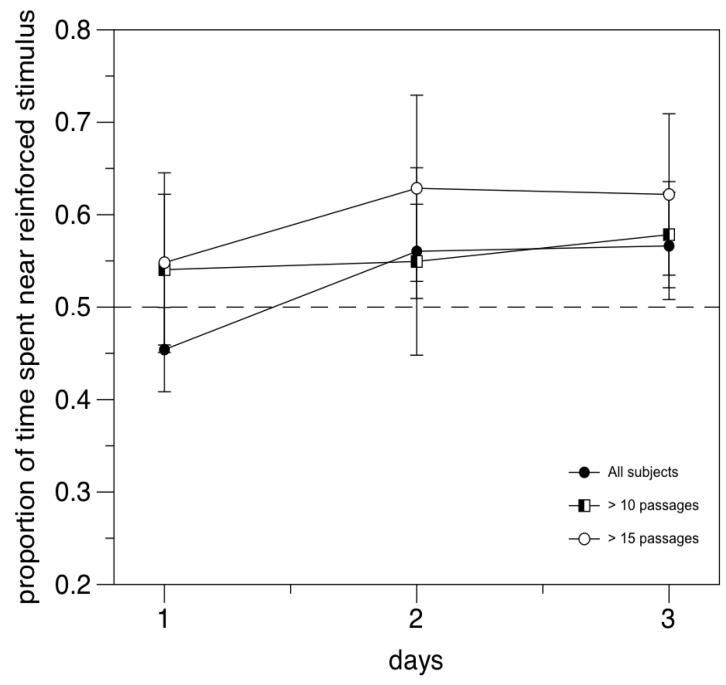
Experiment 1. Performance as a function of stringency of inclusion criteria. Fish performance improved when only subjects with high inter-compartment mobility were included in the analysis. Each point represents the mean of the two daily trials.

With respect to the performance, the proportion of time spent near the reinforced stimulus (average preference in the six trials of individual training) in the whole 24 subject sample is not significantly higher than chance (0.540 ± 0.171; one sample *t*-test, t(23) = 1.148, *p* = 0.263). Preference for the reinforced stimulus increases when we applied the criterion of inclusion of the previous study, at least 10 passages over six trials (N = 8, 0.574 ± 0.136; t(7) = 1.543, *p* = 0.167), achieves statistical significance when only the subjects who moved at least 15 times are included (N = 5, 0.641 ± 0.083; t(4) = 3.801, *p* = 0.019; Figure 5).

A mixed model ANOVA was conducted on subjects that performed at least 10 passages in the six trials (the criterion adopted in previous study). Performance did not significantly vary across the trials (Repeated Measures ANOVA: (F(1,44) = 0.122, *p* = 0.728). No effect of the overall number of movements between compartments (F(1,44) = 2.821, *p* = 0.1) or of the type of conditioned stimulus (F(1,44) = 0.963, *p* = 0.332) was found. No interaction between these variables was significant (all *p* values > 0.585). The conclusions are the same when we included all 24 subjects in the analysis or when we used a more stringent exclusion criterion.

**Figure 6:**
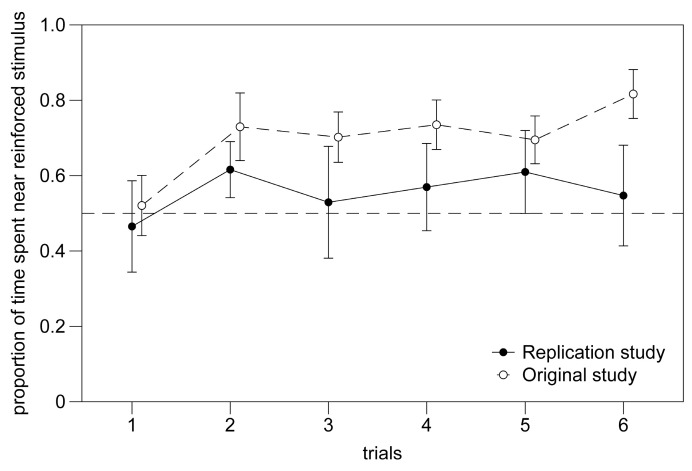
Experiment 1. Performance trend over three days of individual training in this experiment (solid line) and in identical experiment of previous study (dotted line; Santacà et al. [27]).

To compare the two studies, we performed an overall analysis including the subjects with at least 10 passages (this study N = 8; original study N = 12). We found a significant effect of study (Repeated Measures ANOVA: F(1,8) = 6.518, *p* = 0.034; Figure 6), but no effect of the trial (F(1,8) = 1.319, *p* = 0.284) or type of conditioned stimulus (F(1,8) = 0.379, *p* = 0.555). We found the triple interaction “conditioned stimulus x average movements between compartments per trial x study” to be significant (F(1,8) = 6.646, *p* = 0.033). All the other interactions between variables were not significant (all *p* > 0.155).

### 3.2. Experiment 2

Seven out of forty-six subjects (15.21%) died during the experiment; we analyzed the data obtained from the remaining 39 individuals. The mortality rate is significantly lower than in Experiment 1 (chi-square = 11.3318 *p* < 0.001).

In this experiment, we measured time spent in three areas, two choice areas and an intermediate no-choice area. The no-choice area encompasses a portion of both compartments and it was therefore not feasible to measure the number of passages between compartments as we did in the previous experiment. In a large proportion of trials (130/381, 34.1%), subjects scored 100% of the time in one of the two choice areas, meaning that they also did not move between compartments. As a proxy for individual mobility, we used the proportion of trials in which each subject made at least one passage between two adjacent areas. This mobility index ranged from 0.3 to 1.0, with an average of 0.656 ± 0.191.

**Figure 7:**
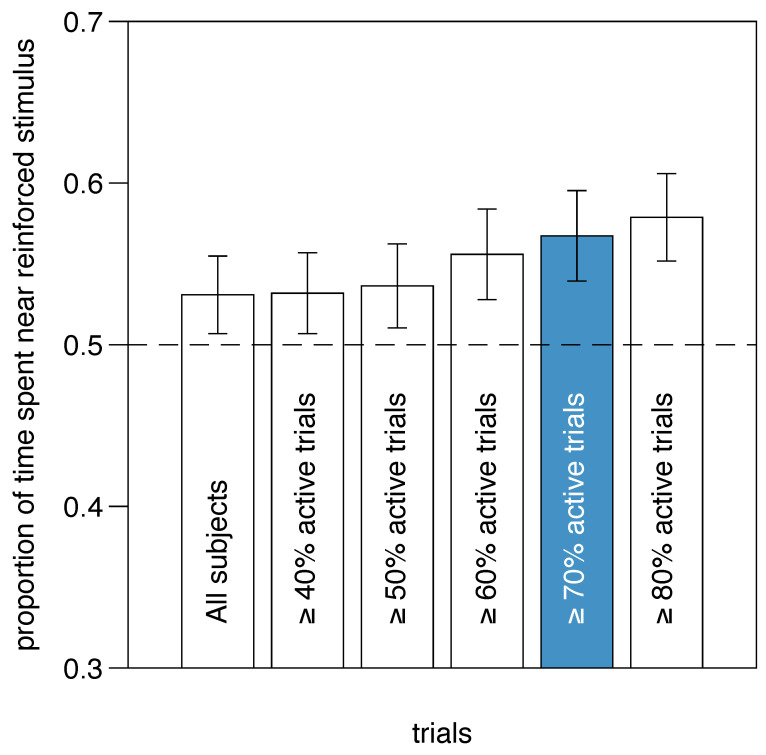
Experiment 2. Learning performance as a function of stringency of inclusion criteria. Inclusion criterion was percentage of trials in which a subject performed at least one passage between the two compartments (i.e., active trials). Performance improves as more selective criteria for inclusion are applied, suggesting that less active larvae may not have learned the task. Given the tradeoff between selectivity and sample size, we used a 70% threshold (highlighted in blue) for further statistical analysis.

We examined the relationship between inter-compartment mobility and learning performance, measured as the proportion of time spent near the reinforced stimulus. Figure 7 shows the average performance in the second half of the training as the inclusion threshold for mobility is varied. There is a significant positive correlation between inclusion threshold and performance (Kendall’s rank correlation; τ = 0.153, *p* = 0.0032), indicating that excluding fewer active larvae improves the average performance level. This suggests that inactive individuals likely failed to learn the task.

Since there is a tradeoff between selectivity and sample size, we decided to set the criterion so as to include in the final sample only subjects with at least 70% of non-inactive trials. Using this criterion, 21 subjects were included in the analysis and 18 were excluded. An overall analysis of the 10 trials did not evidence a statistically significant preference for the sector with reinforced stimuli (one-sample *t*-test: t(20) = 0.561, *p* = 0.581). However, a significant preference for the correct compartment emerged when considering only the second part of the training (t(20) = 2.393, *p* = 0.027). When trials 6 to 10 are analyzed separately for the two sub-procedures, only A sub-procedure was significantly above chance level (one-sample *t*-test: t(12) = 3.444, *p* = 0.005; B sub-procedure: t(7) = 0.599, *p* = 0.568).

**Figure 8:**
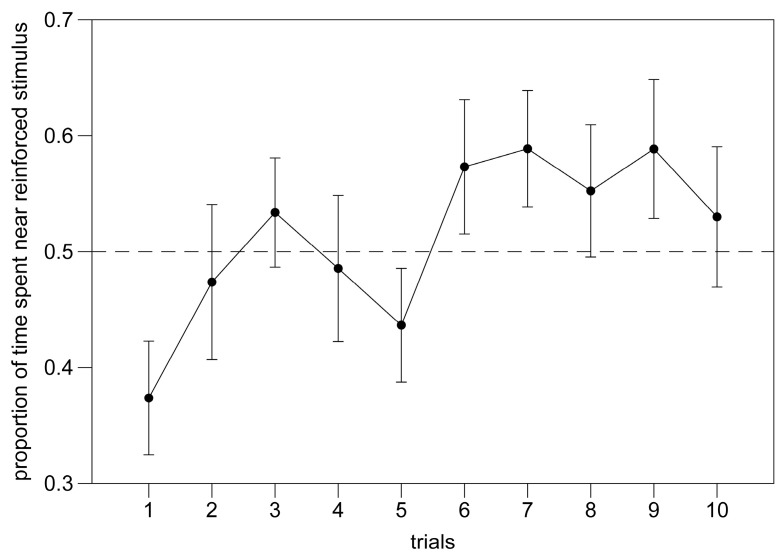
Experiment 2. Performance trend over five days of individual training. A significant preference for the reinforced stimulus emerged in the second part of the training.

A mixed model ANOVA showed no effect of the stimulus pattern on the performance (F(1,206) = 0.011, *p* = 0.916; Figure 8) and no effect of the procedure used (A vs. B sub-procedure; F(1,206) = 0.854, *p* = 0.357). Variation of performance across trials is nearly significant (Repeated Measures ANOVA: F(1,206) = 3.796, *p* = 0.0527). No interaction between these variables was significant (all *p* values > 0.05).

### 3.3. Experiment 3

All subjects survived until one week after the end of the experiment. On average, subjects moved between compartments 20.51 (±12.32) times per trial (range 9.27–43.64).

For technical reasons, data could not be collected for five out of thirty-two subjects in the 12th trial; these were treated as missing values in the analyses. An overall analysis of performance was conducted on the average performance of the second to sixth day of training (trials 3–12). Trial 1 was excluded because the morning session on the first day preceded the first reinforcement, and trial 2 was excluded because the afternoon session followed only a single reinforcement. Although there are instances where learning occurs after a single appetitive or aversive event, such cases are rare. In most species, including zebrafish, a single reinforcement provided through appetitive learning protocols is generally insufficient to induce learning [41,42]. Overall, subjects showed a significant preference for the reinforced stimulus: (mean ± std. dev: 51.8 ± 1.61; one-sample *t*-test, t(9) = 3.363, *p* = 0.008). We did not find a significant correlation between average number of passages between compartments and performance (Pearson’s product-moment correlation, rho(30) = −0.197, *p* = 0.280).

**Figure 9:**
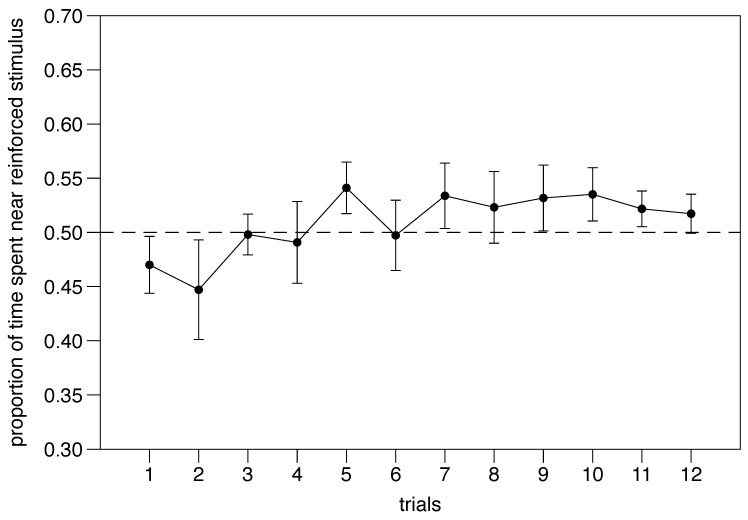
Experiment 3. Performance trend over six days of individual training.

Performance through trials 1–12 was analyzed with a mixed model ANOVA. We found no significant effect of trial (F(1,368) = 1.456, *p* = 0.228; Figure 9), a significant effect of the reinforced stimulus (F(1,368) = 66.646, *p* < 0.001), and a significant trial x stimulus interaction (F(1,368) = 6.209, *p* = 0.013); the number of movements between compartments had no significant effect on the performance (F(1,368) = 0.174, *p* = 0.677). All other interactions were not significant (*p* > 0.296).

## 4. Discussion

Experiment 1 was a replication of the procedure developed by Santacà and colleagues [27]. In this experiment, we confirmed the overall trend observed in the original work: zebrafish larvae, which were repeatedly associated with the release of food upon one stimulus, after five days of training spent significantly more time near this stimulus than near the unreinforced stimulus. However, the outcome of the two experiments differed in many respects and we confirmed the presence of serious limitations in the procedure already apparent in the previous study.

Firstly, this experiment highlighted a problem of very high mortality, with 50% of the subjects (24 out of 48) dying during the course of the experiment. This figure contrasts sharply with a mortality rate of less than 5% in other experiments conducted in the same laboratory (e.g., [43]), but is also significantly higher than that observed in the experiment we replicated (29%). Additionally, the number of larvae with low inter-compartment mobility was found to be very high and significantly greater than that recorded in the original study. The larvae that passed fewer than 10 times from one compartment to the other in total in the six trials (the exclusion criterion adopted in the previous study) accounted for 67% (compared to 43% in the previous study). It is possible that these two phenomena are related. With our procedure, two meals per day were provided. This quantity is the minimum required for larvae of this age. Subjects that happened to be in the non-reinforced compartment at the time of stimuli introduction needed to move into the opposite compartment to consume the reward. Consequently, subjects with poor mobility had a great chance of not passing into the rewarded compartment before the food degraded (usually 10–15 min), hence increasing the risk of dying of starvation.

When evaluating learning performance, in accordance with the previous study, we excluded from the analysis subjects with a low rate of passages. With the 10-passages cutoff used in the previous study, in our experiment the preference for the reinforced stimulus did not reach significance. This could also be due to statistical power issues, since, with this cutoff, only eight subjects out of twenty-four remained in the final sample. A significant preference was observed when a more selective cutoff (at least 15 passages in six trials) was adopted, although in this case, the sample size was further reduced to five. A direct comparison of the two experiments evidenced a significantly lower performance in the replication experiment.

This experiment was conducted using the same equipment and procedures as the previous one, making it unclear what caused the differences observed between the two studies. While there were minor procedural discrepancies between the experiments, it is unlikely that these account for the differences. Such discrepancies include, for instance, the brand of food routinely used to feed larval zebrafish in the two laboratories, the water conditioning system, or the brand of the lighting system employed.

The two experiments also differed in the strain used. While both laboratories routinely employ subjects from a large outbred population, the two stocks originate from different sources and are periodically restocked with individuals obtained from distinct local suppliers. Differences in larval behavior among zebrafish strains are well-documented. Although inter-strain variation in learning capacity is rarely reported, traits such as locomotor activity and boldness frequently differ between strains [44,45]. It is therefore possible that such differences exist between the Padua and Ferrara stocks. Variability in these traits could influence the tendency to move between compartments, affecting the number of effective reinforced trials received by the subjects, and ultimately impacting both mortality and learning rates.

In summary, while we confirmed that larvae can be trained with a fairly limited number of trials to discriminate between two visual stimuli, we found only partial replicability of the procedure. This first experiment also confirmed the presence of serious limitations of this conditioning protocol but, at the same time, we were unable to provide hints on how to overcome these limitations without substantially modifying the procedure. In the second half of this study, we attempted two modifications to the procedure, both aimed at facilitating the passage of larval zebrafish between compartments, in the hope that these changes would lead to a reduction in mortality and an improvement in discrimination learning performance.

In Experiment 2, we made minor adjustments to the apparatus and procedure to address the issues discussed above. The hole separating the two compartments was enlarged (about 10 times larger) to facilitate the passage of the larvae. However, this modification allows a significantly greater transfer of food particles or their odor between compartments, potentially leading to reinforcing the negative stimulus as well. To mitigate this problem, the apparatus was extended, effectively doubling the distance between the two stimuli. The second change stems from the fact that in the absence of water turbulence, the diffusion of food particles and their odor is largely due to the particles remaining on the surface of the water. In fact, due to surface tension, these particles spread across the entire surface of the apparatus within seconds. To limit this phenomenon, we placed a horizontal bar immersed 2 mm into the water at the midpoint of the apparatus, which is supposed to limit the movement of surface particles.

The results of this experiment confirm that larval zebrafish are capable of associating food reinforcement with a specific visual stimulus, although their learning performance was even more modest than in the previous experiment. Performance was higher in subjects that moved between compartments more frequently, suggesting that the number of passages played a critical role in learning in this experiment as well. Notably, the modifications introduced to the original procedure failed to resolve the issue of reduced inter-compartment mobility, which remained low in Experiment 2 as well.

The results of this experiment once again indicate that the larval zebrafish are capable of associating food reinforcement with a specific visual stimulus. However, this procedure also failed to resolve the issues of reduced passage between compartments, which remained low even in this experiment. In this experiment, we observed only a slight increase in the time spent near the reinforced stimulus over the course of the experiment. This was particularly evident among the subjects that passed between compartments more frequently, suggesting that the number of passages plays a critical role in discrimination learning even in this experiment.

Regarding the failure of hole enlargement to solve the problem of low frequency of passages, some video footage shows larvae approaching the central hole, but seemingly bouncing off as if it were impassable. At this age, larvae primarily swim near the water surface, and it is possible that a barrier submerged even just 2 mm below the water surface constitutes a significant obstacle for them.

Mortality was significantly reduced compared to Experiment 1. This progress is probably not solely due to the improvement in mobility between compartments (which remains low even in this experiment), but rather to the use of the new contingency approach. In this experiment, reinforcement was delivered only when the subject was in the compartment with the correct stimulus, increasing the probability of subjects with low mobility to consume the reward.

In this experiment, we also compared two slightly different sub-procedures. In the A sub-procedure, stimuli were continuously present in the experimental setup from the time the larvae were transferred at 7 days post-fertilization (dpf) until the conclusion of the 5 training days. The B sub-procedure stimuli were introduced at the beginning of each trial and removed at the end as in Experiment 1. We found that subjects tested with the A sub-procedure had a better learning performance. This may be related to the poor inter-compartment mobility shown by the subjects in this experiment as well. Subjects tested with the A sub-procedure could move well in advance to the correct compartment since the stimulus remained there for many hours before the trial. In contrast, the subjects of the B sub-procedure at the time of stimulus introduction had a 50% chance of being in the correct compartment and, if not, only 60 min to move to the reinforced sector in time to receive the reinforcement correctly.

In Experiment 3, we adopted a radically different approach. The passage was further widened and we removed the surface barrier used to limit the movement of floating food particles. In this way, however, the food and its odor were free to diffuse. To address this problem, at the time of reinforcement, the two different stimuli were replaced with two identical stimuli (both of the type reinforced for that subject) so that whichever compartment the larva was in, the food was associated only with the correct stimulus.

Even in this experiment, the results obtained at the completion of the training were modest compared to those achieved with the original procedure. The subjects significantly increased their preference over the days and overall showed a significant preference for the reinforced stimulus. However, by the end of the experiment, the preference did not reach 55%, well below the 70% preference observed in the original study [27]. It is unclear why learning was so poor. The most likely hypothesis is that effective discrimination of two stimuli requires the reinforced stimulus to be presented together with the non-reinforced stimulus at the time of reinforcement. The method used in the third experiment is comparable to the successive discrimination procedure, well studied in rats and pigeons (reviewed by Zentall and Clement [46]). In this study, the positive and negative stimuli were presented individually in succession, and only the former was associated with a reward. It has been observed that with this procedure, in many cases, some of the value of the positive stimulus transfers to the negative stimulus with which it is paired. It is possible that this mechanism also acted in our experiment, decreasing the relative preference for the reinforced stimulus during the learning assessment.

In this experiment, the structure of the apparatus led to a substantial increase in the frequency of passages between compartments, averaging 20 per hour. This is a striking contrast to experiments using the original procedure, where approximately half of the subjects were excluded for averaging fewer than one passage per hour. Notably, mortality in this experiment dropped to zero, compared to the exceedingly high levels reported in experiment employing the original procedure (29% [27]; 50% in Experiment 1, this study). While unidentified factors may contribute to this difference, it is plausible that the elimination of mortality is linked to the fact that, in Experiment 3, all subjects received food in every trial while, under the original procedure, less mobile subjects likely only accessed food during trials in which they happened to be in the correct compartment at the time of food release.

In recent years, the replicability of scientific findings and the reproducibility of experimental procedures have become critical topics in the scientific community [47,48]. It is now widely recognized that validating results through subsequent studies is essential, and that replication efforts must closely adhere to the original protocols to avoid introducing confounding factors. Substantial deviations from the original methodologies can lead to erroneous conclusions about non-replicability [49,50,51,52,53]. Following this principle, we replicated the original experiment in a different laboratory while maintaining strict adherence to the initial procedure. Given the significant differences observed between the original findings and their replication, further replications are warranted to investigate the potential causes of these discrepancies.

A second issue that has gained prominence in recent years is the problem of publication bias. The most critical aspect of this phenomenon is that studies with statistically significant results are more likely to be published than those that fail to confirm experimental hypotheses [51,52]. This bias can produce misleading conclusions about a phenomenon, reduce the reproducibility of published results, and lead to flawed outcomes in meta-analyses and systematic reviews. Less attention has been paid to another form of publication bias: the tendency to omit reporting on experimental procedures that prove unsuccessful [53]. The lack of communication about non-functional procedures can lead to repetitive studies of unsuccessful procedures, resulting in unnecessary expenditure of human and financial resources that could otherwise be directed toward more promising avenues. In fields such as medicine and psychology, where developing effective therapeutic strategies is paramount, this form of publication bias can lead to flawed conclusions about the relative validity of different therapeutic approaches. This principle guided our decision to publish the two subsequent experiments, despite the fact that the modified procedures proved less effective than the original one. We believe that reporting these results provides valuable insights into the challenges of refining experimental paradigms and highlights the importance of transparency in methodological development.

## 5. Conclusions

This study confirms with three different methodologies that larval zebrafish are capable of appetitive conditioning a few days after hatching and can associate reinforcement with a specific visual stimulus with ten reinforced trials or less. However, the originally developed procedure is confirmed to have significant limitations, the main one being limited replicability. Since we conducted only a single replication experiment, it is unclear whether this limited replicability is inherent to the methodology or if it is due to differences in laboratory conditions, in the stock, or different timing compared to the original study, or to the unintended introduction of confounding factors not present in the original work. Despite our efforts, we were unable to overcome the procedural issues observed in the original procedure, and both modifications of the procedure that we tested resulted in poorer learning performance. Clearly, further exploration of alternative approaches is necessary to make the procedure efficient and replicable. Achieving this goal is crucial given the importance of the larval zebrafish model for numerous fundamental and applied research areas.

## Data Availability

The original contributions presented in the study are included in the article, further inquiries can be directed to the corresponding author.
